# Caregiver feeding practices for infants and toddlers and their eating behaviors in Singapore

**DOI:** 10.3389/fnut.2025.1540031

**Published:** 2025-03-04

**Authors:** Phaik Ling Quah, Daniel Wei Keong Chan, See Ling Loy, Chengsi Ong, Chai-Hoon Nowel Tan, Michael Yong Hwa Chia, Terence Buan Kiong Chua, Fabian Yap, Mei Chien Chua, Kok Hian Tan

**Affiliations:** ^1^Division of Obstetrics & Gynecology, KK Women’s and Children’s Hospital, Singapore, Singapore; ^2^Duke-NUS Medical School, Singapore, Singapore; ^3^Endocrinology Service, KK Women’s and Children’s Hospital, Singapore, Singapore; ^4^Lee Kong Chian School of Medicine, Nanyang Technological University, Singapore, Singapore; ^5^Department of Reproductive Medicine, KK Women’s and Children’s Hospital, Singapore, Singapore; ^6^Department of Nutrition and Dietetics, KK Women’s and Children’s Hospital, Singapore, Singapore; ^7^Department of Pediatrics, KK Women’s and Children’s Hospital, Singapore, Singapore; ^8^Physical Education & Sports Science, National Institute of Education, Nanyang Technological University, Singapore, Singapore; ^9^Department of Neonatology, KK Human Milk Bank, KK Women’s and Children’s Hospital, Singapore, Singapore

**Keywords:** infant, early childhood, feeding practices, eating behaviors, early life nutrition

## Abstract

**Introduction:**

Research on early childhood caregiver feeding practices and eating behaviors is limited, especially within Asian populations. This study examined these practices across key feeding domains of variety, autonomy, and mealtime setting and timing, stratified by three age groups: 0 to <7 months, 7 to <13 months, and 13 to <36 months.

**Methods:**

A cross-sectional survey of 1,307 caregivers from a multi-ethnic population in Singapore captured demographic data, feeding practices, child eating behaviors, and caregivers’ knowledge, attitudes, and practices. One-way analysis of variance (ANOVA), independent T-tests and the chi-square test were used to assess feeding practices and eating behaviors across age groups.

**Results:**

Regarding dietary variety, 14.8 and 6.1% of infants aged 7 to <13 months were offered three or fewer food groups frequently and daily, respectively. Additionally, 11.9% of infants were receiving processed foods often. At this age, only 1.0% of infants were consuming sugar-sweetened beverages (SSBs) often, while 2.0% consumed them daily. Among older children (aged 13 to <36 months), 8.1% were offered a limited variety of three food groups, while 4.5% were offered fewer than three. In contrast, a significantly higher proportion frequently consumed processed foods (24.0%) and sugar-sweetened beverages (25.2%; *p* < 0.05). In terms of autonomy, only 75.4% of infants (7– < 13 months) and 89.5% of older children (13– < 36 months) were able to self-feed. Caregivers of older children (13– < 36 months) were less likely to recognize hunger and satiety cues compared to those of infants (0–< 13 months; *p* < 0.05). Older children (13– < 36 months) also more frequently required special mealtime settings (36.6%), viewed screens during meals (29.9%), and were less likely to be offered post-midnight meals nightly (22.6% compared to infants; 70.3%; 0–< 13 months; *p* < 0.05).

**Conclusion:**

These findings underscore the need for culturally tailored educational interventions to improve suboptimal feeding practices in children under three in Singapore’s multiethnic population.

## Introduction

Caregiver feeding practices and early childhood eating behaviors are fundamental for a child’s healthy growth, development, and shaping of life-long (1, 2) healthy eating behaviors and dietary patterns (3). Infants and toddlers are key targets (4) with early childhood feeding practices implicated in programming lifelong risks for non-communicable diseases like obesity, diabetes, heart disease, and certain cancers (5, 6). Caregivers play a central role in shaping eating behaviors through optimal feeding practices early in life, determining what, when, and how much children eat (7). The literature identifies four key domains that significantly impact early childhood nutrition: variety (8, 9), autonomy (10–12), setting (13–15), and timing (16–21), all of which help shape children’s dietary habits and overall health.

Early exposure to diverse flavors and textures during the appropriate timing of solid food introduction (16) fosters the acceptance of healthy foods (22) and builds habits that often persist into later life (23), highlighting the importance of varied food access in early childhood (8, 9). Dietary variety is associated with a better nutritional profile (24, 25) essential to achieve adequate coverage of macro- and micronutrient needs (8), and reduced food fussiness at later ages (26). In addition, an authoritative feeding style which promotes child autonomy within structured boundaries, supports optimal self-regulation in eating (12). Evidence suggests that this approach where caregivers decide when, where, and what food is offered, while children decide if and how much to eat, best fosters children’s ability to recognize hunger and fullness cues, consumption of a variety of food and achieve a nutritionally adequate diet (27). In contrast, relying solely on parental cues may undermine children’s self-regulation of food intake, increasing the risk of overeating and weight gain (10).

Children’s eating behaviors are also shaped within the family context, with the mealtime setting playing a critical role. Parental monitoring and consistent family mealtime routines during early childhood foster a supportive food environment that can significantly improve children’s overall diet quality (28). Studies show that frequent family meals are linked to healthier eating behaviors in children, including higher fruit and vegetable intake (29), and reduced risk of disordered eating (30). It also matters whether parents eat the same food as their children, as role-modeling of this behavior has been linked to lower levels of food fussiness, greater food enjoyment in children (13) and heathier dietary intake in children (31, 32). In contrast, factors like eating away from the dining table and mealtime distractions (e.g., screen time viewing) are associated with feeding problems (14), higher intake of ultra processed foods (15), sweets and desserts, sugar sweetened beverage consumption in young children (33), higher total energy intake and an increased risk for obesity (34).

The timing of child feeding practices includes critical components such as, the pace at which a child eats (18–20, 35), and the schedule of meal timings (36). The World Health Organization recommends exclusive breastfeeding for the first 6 months of life, with continued breastfeeding for up to 2 years or beyond (16). Complementary foods should be introduced in a timely manner, starting at 6 months of age. Some evidence suggests that introducing complementary foods too early (at or before 4 months) may increase the risk of childhood overweight (37) compared to introduction at 4–6 months or after 6 months. Eating rate has also been identified as a marker for future weight and fat gain (20) where in preschoolers, eating rate at age 4 correlated positively with weight and adiposity at age 6, independent of maternal weight, linking rapid eating with excessive weight gain (19). Furthermore, recent evidence indicates that the timing of food intake may influence body weight regulation (36). Observational studies in children indicate that meal timing, particularly consuming a large proportion of energy later in the day and into the evening, may negatively affect weight status. For example, studies have shown that decreasing nighttime feedings for infants aged 6–12 months aids in managing calorie intake, supporting healthy growth (38, 39). Furthermore, infants consuming more calories at night (after 7 pm) than during the day at 12 months have been associated with greater body fat gain and an increased risk of overweight and obesity by 24 months (40).

Despite substantial evidence, implementing healthy feeding practices in multi-ethnic Asian settings continues to face persistent challenges and gaps, often influenced by factors such as ethnicity, cultural misconceptions (41, 42) and the role of primary caregivers contributing to suboptimal practices (43, 44) that may differ from those seen in Western contexts. Building on these identified gaps, there is a need to further investigate and understand caregiver feeding practices and children’s eating behaviors in these populations. Thus, this study aims to describe caregiver feeding practices, and eating behavior in children in Singapore, focusing on three distinct age groups: infants aged 0 to <7 months, infants aged 7 to <13 months, and toddlers aged 12 to <36 months. The study emphasizes key feeding dimensions, including variety, autonomy, setting, and timing, within a multi-ethnic Asian population.

## Methods

### Study sample

This study employed a cross-sectional design, enlisting caregivers as part of the Integrated Variety Autonomy Setting Timing feeding practices study in infants’ and toddlers’ (I-VAST) study. A convenience sampling method was employed to select caregivers from various study sites in Singapore. Eligible caregivers were individuals who were either Singaporean or permanent residents, caring for children aged 0 to below 36 months of age, and were able to understand English. Caregivers were excluded from the study if their children did not fall within the specified age range or if they were unable to understand English. Age-appropriate surveys were developed, tailored to accommodate each specific age group (Supplementary Figure S1). Responses were collected from children in three age groups: (1) 0–<7 months, (2) 7–<13 months, and (3) 13–<36 months. The survey was distributed via a QR code, targeting eligible caregivers with children aged 0 to <36 months. Participation in the survey was considered as implied consent upon accessing the QR code and completing the survey. All participants will access the survey electronically through the FormSG platform.^1^ The research procedures for this study received formal approval for an exempt review from the SingHealth Centralized Institutional Review Board (CIRB Ref No.: 2023–2,539). All the authors and caregivers of the children surveyed have provided consent for the publication of this manuscript.

### Data collection

Between October 2023 and April 2024, a total of 1,307 responses were collected in total. The online questionnaire was designed with adaptive functionality, ensuring that only age-appropriate questions appeared based on the caregiver-selected child’s age group. This streamlined the questionnaire, presenting caregivers with relevant items tailored to their child’s age group. The survey gathered data on the child’s baseline characteristics, feeding and eating practices pertaining to the domains of variety, autonomy, setting, and timing, as well as the knowledge, attitudes, and practices of caregivers. Caregivers provided their demographic characteristics and reported on their child’s feeding and eating behaviors. They were asked to recall their child’s most recent typical week when responding to the survey. The survey questionnaire was developed and reviewed by members of the College of Pediatrics and Child Health Singapore-Integrated Platform for Research in Advancing Maternal & Child Health Outcomes (CPCHS-IPRAMHO) Feeding and Eating in Infants and Young Children Study Group. The workgroup reached a consensus on four key domain areas, comprising ‘Variety’, ‘Autonomy’, ‘Setting’, and ‘Timing’ domains that parents and caregivers should prioritize to foster healthy eating behaviors and habits in infants and young children (45). These areas include promoting food variety, encouraging eating autonomy, creating distraction-free family mealtimes, and establishing regular daytime eating schedules. The development of the survey questionnaire went through an expert review and thorough literature review, establishing its content validity. The survey took 10–15 min in total duration to complete.

### Caregiver feeding practices

Caregivers were asked to report whether they had introduced solids or liquids other than milk to their child, and if so, at what age. In the context of the questionnaire, “milk” refers to either breast milk or formula. Based on a national breastfeeding survey conducted in 2011, we assume that approximately 80% of infants aged 0 to <7 months are still receiving breast milk. However, by 7 months and older, the majority of children are likely to be receiving formula milk (46). Additionally, caregivers were asked to indicate what percentage of their child’s total food intake was made up of milk feeds. To assess food preparation, caregivers were asked if their child ate the same food as the rest of the family, and if the meals were prepared with added sugar or salt. Parental perceptions and practices regarding child feeding and eating were assessed through four key questions. Caregivers were asked if they found it difficult to get their child to eat, had concerns about their child’s weight, and whether mealtimes were generally challenging. They also ranked their priorities when selecting and providing food during meals.

### Assessment of variety

The survey assessed the child’s exposure to different food groups (e.g., starches and grains, proteins, fruits, and vegetables), including a variety of textures and flavors. To capture the intake of discretionary foods, caregivers were also asked to report the frequency (never, occasionally, a few times a month, once a week, a few times a week, or daily) with which their child was offered packaged, canned, or processed foods and sugar-sweetened beverages (SSBs; e.g., sugary drinks, malt drinks, yogurt drinks).

### Assessment of autonomy

To assess feeding autonomy and the child’s mealtime experience, caregivers were asked if the child had begun feeding themselves: using finger foods for children aged 7 to under 13 months, and using their hands or utensils for children aged 13 to under 36 months. They were also asked if their child enjoyed mealtimes, and if they believed their child was consuming sufficient amounts of milk and solids. To evaluate caregiver attentiveness and responsiveness to the child’s hunger and satiety cues from birth, caregivers were asked whether they could recognize when their child was hungry or full, and whether they encouraged their child to finish all the food or milk provided.

### Assessment of setting

To assess if children were being provided an appropriate environment for a nurturing and secure feeding and eating environment, caregivers were asked if they had to do anything special to help their child feed (such as using distractions with toys), if the child was seated on a highchair during meals (only for children 0 to below 7 months old, and 7 months to below 13 months) and if they were having their meals seated together with the family at the table (only for children 13 months to less than 36 months old). The use of screen time during meals was assessed by asking parents if they engaged in screen time while eating in the presence of the child, and if the child had meals in front of a screen or an electronic device.

### Assessment of timing

To evaluate whether caregivers were gradually transitioning from round-the-clock feeding to a more structured daytime feeding as their child neared 1 year old, caregivers were asked if their child had regular milk or solid feeding times (only for children aged 7 to 12 months and 13 to 35 months). They were also asked whether their child was still being given milk between midnight and 6 a.m. To assess the child’s eating behavior, particularly the speed of eating, caregivers reported how long their child typically took to complete a milk feed or a main meal.

### Statistical analyses

Continuous datasets that were normally distributed are presented as the mean and standard deviation (SD). A one-way analysis of variance (ANOVA) or independent t-test was used to compare continuous variables across age groups, while the chi-square test was employed to analyze differences in categorical variables. Sex- and age-specific body mass index (BMI) z-scores were derived using the WHO references for children 0–5 years of age (47). Statistically significant results were determined at 2-sided *p <* 0.05. All analyses were performed using STATA software version 13 (StataCorp, College Station, United States).

## Results

### Characteristics of study population

The proportion of respondents who were the main caregivers was 93.1% in the youngest age group (0 to <7 months), 91.8% in the 7 to <13 months group, and 87.9% in the 13 to <36 months group, with a majority being mothers. With regards to ethnic distribution, Chinese respondents were the largest proportion across the age groups: 89.1% in the 0 to <7 months group, 80.5% in the 7 to <13 months group, and 83.3% in the 13 to <36 months group. The average age of caregivers ranged from 33.9 (SD: 4.6) years in the 7 to <13 months group to 35.0 (5.9) years in the 13 to <36 months group. The average age of children was 3.8 (SD: 2.5) months in the 0 to <7 months group, 9.1 (SD: 1.6) months in the 7 to <13 months group, and 23.4 (SD: 6.9) months in the 13 to <36 months group. Childcare attendance was highest among children in the 13 to <36 months group (69.2%) compared to the youngest group (18.1%; Table 1). There were no statistically significant differences found with the other characteristics presented.

**Table 1:** Caregiver characteristics among children by age group.

	0 to <7 months; *n* = 404	7 to <13 months; *n* = 267	13 to <36 months; *n* = 636	*p* value
Caregiver characteristics				
Main caregiver, n (%)				
Yes	376 (93.1)	245 (91.8)	559 (87.9)	0.015
Relationship with main caregiver, n (%)				
Mother	319 (79.0)	206 (77.2)	480 (75.5)	0.532
Father	74 (18.3)	58 (21.7)	142 (22.3)	
Grandparents	10 (2.5)	2 (0.8)	12 (1.9)	
Others	1 (0.2)	1 (0.3)	2 (0.3)	
Ethnicity, n (%)				0.003
Chinese	360 (89.1)	215 (80.5)	530 (83.3)	
Malay	32 (7.9)	39 (14.6)	64 (10.1)	
Indian	6 (1.5)	11 (4.1)	21 (3.3)	
Others	6 (1.5)	2 (0.8)	21 (3.3)	
Highest education, n (%)				0.106
University	325 (80.45)	212 (79.4)	522 (82.08)	
Post-secondary	72 (17.82)	54 (20.2)	98 (15.41)	
Primary	7 (1.73)	1 (0.37)	16 (2.52)	
Age (years), mean (SD)	34.2 (5.9)	33.9 (4.6)	35.0 (5.9)	0.007
Firstborn, n (%)	265 (65.59)	162 (60.7)	415 (65.25)	0.510
Child characteristics				
Male, n (%)	202 (50.0)	137 (51.3)	344 (54.1)	0.412
Age (months), mean (SD)	3.8 (2.5)	9.1 (1.6)	23.4 (6.9)	0.001
Childcare attendance, n (%)				
Yes	73 (18.1)	92 (34.5)	440 (69.2)	0.001
z-BMI (z-scores), mean (SD)	0.13 (1.55)	0.29 (1.5)	0.28 (1.6)	0.435
Overweight/Obese (>1SD), n (%)	63 (26.0)	35 (25.6)	124 (30.9)	0.292

Data in Table 1 was presented as frequency (%) for categorical data, or mean (standard deviation) for continuous data. Missing variables: Child age: 0 to < 7 months, *n* = 11; 7 to < 13 months, *n* = 4; 13 to < 36 months, *n* = 12; child z-BMI (*n* = 257).

### Caregiver feeding practices across the different age groups

Caregiver feeding practices differed significantly by age groups (all *p* < 0.05), with the exception of parents’ mealtime first priority ranking (convenience, growth, establishing eating behaviors, or providing nutritious meals; Table 2). The highest proportions of children on solids were in the 7 to <13 months (94.4%) and 13 to <36 months (96.4%) groups, with average starting ages of 6.1 (SD: 0.83) and 6.8 (SD: 2.67) months, respectively. Most 0 to <7 month olds (85.1%) were still receiving over 90 to 100% of their feeds from milk. Among 7 to <13 month olds, 49.4% received less than 60 to 90% of their feeds from milk, while 74.2% of 13 to <36 month olds received between more than 10 to 60% of their feeds from milk.

**Table 2:** Feeding practices across three age groups.

Caregiver feeding practices	0 to <7 months; *n* = 404	7 to <13 months; *n* = 267	13 to <36 months; *n* = 636	*p* value
Solid food intake, n (%)				
Yes	72 (17.8)	252 (94.4)	613 (96.4)	0.015
Age of onset of solid food intake (years), mean (SD)	5.3 (0.96)	6.1 (0.83)	6.8 (2.67)	0.001
% milk feeds, n (%)				0.001
0–10%	15 (3.8)	5 (2.4)	78 (14.0)	
>10–60%	10 (2.5)	99 (48.2)	415 (74.2)	
<60–90%	34 (8.6)	101 (49.4)	66 (11.8)	
>90–100%	337 (85.1)	0 (0.0)	0 (0)	
*Adds sugar or salt to food, n (%)	5 (6.9)	16 (6.4)	369 (60.2)	0.001
*Offers child same food as family, n (%)	–	45 (21.9)	390 (69.8)	0.001
Mealtime struggles				
Yes, n (%)	45 (11.1)	43 (16.1)	187 (29.4)	0.001
Concerned about child weight, n (%)				
Yes-Overweight	29 (7.2)	19 (7.1)	47 (7.4)	0.613
Yes-Underweight	77 (19.1)	56 (21.0)	151 (23.7)	
Caregiver feeding priority, n (%)				0.004
Child to self-feed	118 (29.2)	84 (31.5)	156 (24.5)	
Child to finish food	50 (12.4)	36 (13.5)	124 (19.5)	
Child to eat enough	225 (55.7)	145 (54.3)	333 (52.4)	
Child to eat quickly	11 (2.7)	2 (0.7)	23 (3.6)	
Ranked first priority for mealtimes, n (%)				0.249
Convenience	115 (39.7)	81 (33.5)	171 (32.3)	
Growth	48 (16.6)	51 (21.1)	88 (16.6)	
Establishing eating behaviors	43 (14.8)	39 (16.1)	88 (16.6)	
Providing nutritious meals	84 (28.9)	71 (29.3)	182 (34.5)	

Missing data: % milk feeds: 0 to < 7 months (*n* = 8), 7 to < 13 m (*n* = 62), 13 to < 36 m (*n* = 77); *offers child same food as family: 7 to < 13 m (*n* = 62), 13 to < 36 m (*n* = 77); *Only in children receiving solid foods: 0 to < 7 m (*n* = 72), 7 to < 13 m (*n* = 252), 13 to < 36 m (*n* = 613).

Caregivers were more likely to add salt or sugar to a child’s food in the older age group of 13 to <36 months (60.2%), compared to 7 to <13 months (6.4%) and 0 to <7 months (6.9%). Caregivers of 13 to <36 month olds were also more likely to offer the same foods as the family (69.8%), compared those of 7 to <13 month olds (21.9%). Mealtime struggles increased with age: 11.1% in the 0 to <7 months group, 16.1% in the 7 to <13 months group, and 29.4% in the 13 to <36 months group. Across all age groups, more than half of caregivers prioritized ensuring their child ate enough. Caregivers of 7 to <13 month olds were most likely to prioritize self-feeding (31.5%), while those of 13 to <36 month olds prioritized getting their child to finish all their food (19.5%) and eat quickly (3.6%). Convenience ranked highest as the mealtime priority (32.3–39.7%), followed by nutritious meals (28.9–34.5%), child growth (16.1–21.1%), and establishing eating behaviors (14.8–16.6%) across all age groups (*p* > 0.05; Table 2).

### Feeding practices in the variety domain

All feeding practices in the variety domain in Table 3 were significantly different across the age groups (all *p* < 0.05). Children aged 13 to <36 months were most likely to receive foods containing all four major food groups (87.4%), compared to the younger age groups (7 to <13 months: 79.1% and 0 to <7 months: 75.0%). A higher proportion of 7 to <13 month olds were offered varied textures (93.4%) and flavors (92.7%), compared to the older group of 13- < 36 months olds (textures: 78.5% and flavors: 89.3%). In infancy (0 to <7 months), 25.0% were offered fewer than four major food groups, while 30.5% were offered processed foods and 12.3% consumed SSBs. The highest proportion of children frequently consuming processed foods (24.0%) and SSBs (25.2%) were in the oldest age group (13 to <36 months), compared to younger age groups (Table 3).

**Table 3:** Variety domains across three age groups.

*Food variety domains	0 to <7 months; *n* = 72	7 to <13 months; *n* = 252	13 to <36 months; *n* = 613	*p* value
Number of food groups offered, n (%)				0.013
4	6 (75.0)	155 (79.1)	473 (87.4)	
3	1 (12.5)	29 (14.8)	44 (8.1)	
<3	1 (12.5)	12 (6.1)	24 (4.5)	
Offers child a variety of textures, n (%)				
Yes	–	522 (93.4)	161 (78.5)	0.001
Offers child a variety of flavors, n (%)				
Yes	56 (14.1)	190 (92.7)	499 (89.3)	0.001
*Offers child processed food, n (%)				0.001
Never	50 (69.5)	138 (54.8)	155 (25.3)	
Occasionally	8 (11.1)	78 (30.9)	288 (46.9)	
Often	14 (19.4)	30 (11.9)	147 (24.0)	
Daily or most days a week	0 (0)	6 (2.4)	23 (3.8)	
Offers child sugared beverages, n (%)				0.001
Never	347 (87.7)	180 (90.5)	177 (32.6)	
Occasionally	25 (6.3)	13 (6.5)	207 (38.1)	
Often	8 (2.0)	2 (1.0)	137 (25.2)	
Daily or most days a week	16 (4.0)	4 (2.0)	22 (4.1)	

Missing: Food groups offered: 0 to < 7 m (*n* = 64), 7 to < 13 m (*n* = 64), 13 to < 36 m (*n* = 77); offers child a variety of textures: 7 to < 13 m (*n* = 62), 13 to < 36 m (*n* = 77); offers child a variety of flavors: 0 to < 7 m (*n* = 8), 7 to < 13 m (*n* = 62), 13 to < 36 m (*n* = 77); offers child sugared beverages: 0 to < 7 m (*n* = 8), 7 to < 13 m (*n* = 68), 13 to < 36 m (*n* = 93). *Only in children receiving solid foods: 0 to < 7 m (*n* = 72), 7 to < 13 m (*n* = 252), 13 to < 36 m (*n* = 613).

### Feeding practices in the autonomy and satiety domain

All practices differed significantly by age group, except for the enjoyment of food and the caregiver’s encouragement for the child to finish all food or milk. A higher proportion of 13 to <36 month olds were self-feeding (89.5%) compared to 7 to <13 month olds (75.4%). Overall, the likelihood of identifying hunger and satiety cues was lowest in caregivers of n children in the oldest age group (13 to <36 months). Caregivers of children aged 0 to <7 months were more likely to perceive their child as eating enough (91.5%) and to recognize hunger cues (95.5%), compared to caregivers of children in older age groups. In contrast, caregivers of children aged 7 to <13 months were most likely to identify satiety cues (91.8%; Table 4).

**Table 4:** Autonomy and satiety domains across three age groups.

Autonomy and satiety domains	0 to <7 months; *n* = 404	7 to <13 months; *n* = 267	13 to <36 months; *n* = 636	*p* value
^*^Child self-feeds, n (%)	–	144 (75.4)	470 (89.5)	0.001
Child enjoys ^#^food, n (%)	363 (90.5)	246 (94.3)	496 (91.3)	0.186
Child consumes sufficient ^#^food, n (%)	367 (91.5)	217 (83.1)	447 (82.3)	0.001
Caregiver identifies hunger cues, n (%)	386 (95.5)	242 (90.6)	555 (87.3)	0.001
Caregiver identifies satiety cues, n (%)	365 (90.4)	245 (91.8)	545 (85.7)	0.01
Caregiver encourages child to finish food/milk, n (%)	296 (74.8)	150 (75.4)	420 (77.4)	0.778

Missing data: child self-feeds: 7 to < 13 months (*n* = 61), 13 to < 36 months (*n* = 88); child enjoys food: 0 to < 7 months (*n* = 3), 7 to < 13 months (*n* = 6), 13 to < 36 months (*n* = 93); Child consumes sufficient food: 0 to < 7 months (*n* = 3), 7 to < 13 months (*n* = 6), 13 to < 36 months (*n* = 96); Caregiver encourages child to finish food/milk: 0 to < 7 months (*n* = 8), 7 to < 13 months (*n* = 68), 13 to < 36 months (*n* = 93). #refers to milk and solid feeds; *Only in children receiving solid foods: 0 to < 7 m (*n* = 72), 7 to < 13 m (*n* = 252), 13 to < 36 m (*n* = 613).

### Feeding practices in the setting and timing domain

All feeding practices in these domains significantly differed among the age groups (*p* < 0.05; Tables 5, 6). Children aged 13 to <36 months were most likely to need a special mealtime setting (36.6%), use screens during meals (29.9%), and have caregivers using screens during meals (29.1%), compared to the younger age groups (all *p* < 0.05). A higher proportion of children aged 7 to <13 months ate in a highchair at the table (88.5%) compared to those aged 0 to <7 months (62.5%), while the highest proportion of children who sat at the table with the family during meals times were aged 13 to <36 months olds (64.6%; Table 5). Infants aged 0 to <7 months had a longer average milk feeding duration [17.4 (SD: 11.0)] minutes compared to 7 to <13 month olds [12.3 (SD: 11.5)] minutes. However, 13 to <36 month olds had the longest solid feeding duration [27.3 (SD: 14.3 min)], and were more likely to follow a regular eating schedule (89.5%), and least likely to have post-midnight feeds (46.5%), compared to the younger age groups (Table 6).

**Table 5:** Setting domains across three age groups.

Setting domain	0 to <7 months; *n* = 404	7 to <13 months; *n* = 267	13 to <36 months; *n* = 636	*p* value
Child requires special mealtime setting, n (%)	54 (13.4)	58 (21.7)	(36.6)	0.001
^*^Child eats at table in highchair, n (%)	45 (62.5)	223 (88.5)	71 (11.5)	0.001
^*^Child eats at table with family, n (%)	3 (4.2)	6 (2.4)	396 (64.6)	0.001
Child has meals while engaged with screens, n (%)	2 (2.8)	12 (4.8)	183 (29.9)	0.001
Caregiver uses screens while eating in child’s presence, n (%)	101 (25.0)	43 (16.1)	185 (29.1)	0.001

Missing data: Child eats at table in highchair: 0 to < 7 months (*n* = 3), 7 to < 13 months (*n* = 9), 13 to < 36 months (*n* = 88); Child eats while engaged with screens: 7 to < 13 months (*n* = 9), 13 to < 36 months (*n* = 88); *Only in children receiving solid foods: 0 to < 7 m (*n* = 72), 7 to < 13 m (*n* = 252), 13 to < 36 m (*n* = 613).

**Table 6:** Timing domains across three age groups.

Caregiver characteristics	0 to <7 months; *n* = 404	7 to <13 months; *n* = 267	13 to <36 months; *n* = 636	*p* value
Milk feed duration (minutes), mean (SD)	17.4 (11.0)	12.3 (11.5)	–	0.001
*Solid feed duration (minutes), mean (SD)	17.8 (13.4)	19.4 (11.0)	27.3 (14.3)	0.001
Child has regular eating schedule, n (%)	–	169 (82.4)	500 (89.5)	0.019
Child offered post-midnight meals, n (%)				0.001
Never	37 (9.2)	65 (24.4)	340 (53.5)	
Occasionally	36 (8.9)	47 (17.6)	96 (15.1)	
Often	47 (11.6)	23 (8.6)	56 (8.8)	
Nightly or most nights/week	284 (70.3)	132 (49.4)	144 (22.6)	

Missing value: Milk feed duration: 0 to < 7 months (*n* = 3), 7 to < 13 months (*n* = 6), 13 to < 36 months (*n* = 88); solid feed duration: 0 to < 7 months (*n* = 24), 7 to < 13 months (*n* = 71), 13 to < 36 months (*n* = 111); Child has regular eating schedule: 7 to < 13 months (*n* = 62), 13 to < 36 months (*n* = 59); Solid feed duration: 0 to < 7 months (*n* = 13), 7 to < 13 months (*n* = 7), 13 to < 36 months (*n* = 1). *Only in children receiving solid foods: 0 to < 7 m (*n* = 72), 7 to < 13 m (*n* = 252), 13 to < 36 m (*n* = 613).

## Discussion

This study is the first to comprehensively describe feeding habits of infants and toddlers in Asia across key feeding domains. Mealtime challenges increased with age, affecting a significant proportion of caregivers and children. One-third of caregivers reported difficulties in feeding toddlers. Dietary variety was limited for 20% of infants (0–12 months) and 12.6% of toddlers (13–35 months). Processed food consumption began early, with over one-third of infants being offered such foods and one in eight consuming sugar-sweetened beverages (SSBs). By toddler age, over half consumed both processed foods and SSBs. Feeding autonomy issues were also prominent: 24.6% of infants (7–12 months) and 10.5% of toddlers struggled with self-feeding. Additionally, many caregivers of toddlers were less attuned to their children’s hunger and satiety cues. Toddlers were also more likely to require specific mealtime settings, use screens during meals, and continue post-midnight feedings. Our data further indicate that caregivers’ main priority during meals is ensuring that their child eats enough. While this priority decreases as the child grows older, a greater emphasis is placed on children finishing their meals and eating quickly. Across all age groups, caregivers consistently ranked convenience as their main priority during mealtimes, followed by the provision of nutritious meals. In contrast, promoting child growth and fostering healthy eating behaviors were given lower priority. These findings underscore the need for targeted efforts to promote healthier eating habits among young children in Singapore.

Currently, the only other local cohort examining feeding practices within Singapore is the Growing Up in Singapore Towards healthy Outcomes (GUSTO) study, which reported variations in feeding practices, but focused solely on infants at 9 and 12 months of age and limited feeding practices (41). Compared to our findings, nearly 70% of infants in the GUSTO cohort at 9 and 12 months of age were reported to consume juices and sweetened drinks (41), representing a significantly higher proportion than observed in this study. However, it is important to note that the GUSTO study captured the intake of fruit juices, which could have included 100% fruit juice or sugar-sweetened juices, whereas our study specifically focused only on sugar-sweetened beverages. This difference in categorization may explain the inflated proportions observed between the two studies. The high consumption of SSBs among toddlers in this study is consistent with findings from another local study from the Singapore population, which found that nearly 60% of 18-month-old children consumed SSBs. These beverages predominantly included sweetened drinks such as both carbonated and non-carbonated options, prepackaged fruit juices with added sugars, and sweetened soy milk (48). In terms of diet variety, children aged 12 to <36 months in our study demonstrated better food group variety compared to their Western counterparts aged 12 to <24 months in the 2016 US Feeding Infants and Toddlers Study (FITS) (49), where 20% did not consume any fruits or vegetables. However, in the United Arab Emirates (UAE)—an economically advanced nation similar to Singapore—a study of children aged 6 months to 2.5 years found that most did not meet the recommended daily or weekly intake of essential food groups (50). Notably, toddlers had a higher weekly median intake of chips and sweets compared to fruits and vegetables (50). Likewise, in our Singapore cohort of children aged 12 to <36 months, while 87.4% received food from essential groups, over 70% consumed processed foods. Feeding practices in the Asia-Pacific region, as shown in both rural and urban areas of China, India, and Indonesia in children under 5 years old, consistently reflect low dietary variety (51). Nutrient-dense, protein-rich foods, fruits, and vegetables are less commonly provided, with a reliance on staples such as rice, cereals, and noodles. A significant proportion of children, particularly in India and Indonesia, consumed energy-dense, nutrient-poor snacks and sugary drinks (51). Similarly, reports from Pakistan indicate low dietary diversity in complementary foods for infants aged 6 to 9 months (52). Collectively, these findings highlight a concerning trend: many parents offer less nutritious food and beverage options as their children enter toddlerhood. This practice can negatively impact overall diet quality early on, setting patterns that may adversely affect children’s long-term health and weight status.

The development of emerging child feeding autonomy necessitates caregiver support to offer the foundation and structure needed to establish healthy habits and routines (53). This support includes engaging in reciprocal nurturing feeding practices, such as attentively responding to a child’s hunger and satiety cues, creating a conducive mealtime environment, and minimizing distractions during meals (54). These practices further encourage children to develop preferences for healthy foods and beverages and promote autonomous eating (53). In this study, caregivers of toddlers were observed to be less responsive to recognizing hunger and satiety cues. Concordant with our study observations, Leahy et al. reported that as children grow older, typically between the ages of 3 to 5 years, there is a noticeable decline in responsive feeding practices (55). In the context of the culture in Singapore, caregivers may be less attuned to a child’s hunger and satiety cues at older ages, as approximately 70% of children at 2 years old are already enrolled in childcare centers (56) where feeding practices might not be as individualized as those in a home setting. With respect to child autonomy during mealtimes, our study found that approximately 75% of infants aged 7 to <13 months were able to self-feed. This rate is notably higher than that reported in an Asian cohort from China, where only 10% of infants aged 6–11 months could self-feed (57). It also surpasses findings from a Western population study in the United Kingdom, where 55% of infants aged 8–12 months were reported to self-feed (58). Western cultures generally promote higher rates of infant self-feeding due to norms emphasizing early independence and self-reliance. Conversely, many Asian cultures adopt more authoritarian feeding approaches, with lower responsiveness but greater control (59). Feeding practices are influenced by cultural norms and societal influences, which vary across regions. Singapore’s diverse cultural environment likely contributes to a combination of traditional and modern feeding practices, reflected in these findings.

Our data also revealed that mealtime struggles increased as children transitioned from infancy to toddlerhood. In this age group, there was also a greater need for specific mealtime settings, and an increase in screen use during meals. This underscores the importance of establishing healthy eating practices early, as failing to do so may contribute to suboptimal eating behaviors later in life (22, 60, 61). Meta-analyses confirmed that a greater quantity of screen time is associated with an increased risk of obesity (62, 63). Furthermore, the increase in screen time in children has been linked to poor eating behaviors, such as a preference for energy-dense (64), nutrient-poor foods (65), reduced consumption of fruits and vegetables (66), and a higher intake of SSBs (67). Interestingly, screen use by caregivers during meals was more prevalent across all age groups, with the highest incidence observed among those with toddlers. While physically present, caregivers’ phone use often results in distraction and reduced responsiveness to their children’s needs (68). Myruski *et al.* found that parental phone use during mealtimes is linked to less healthy feeding practices, including reduced positive modeling, and increased negative behaviors such as emotional regulation issues or pressuring children to eat (68). Our findings emphasize the need for targeted interventions and educational programs to promote responsive feeding practices among caregivers early in childhood, aiming to prevent escalating mealtime struggles at later ages.

When examining the timing domain in this study, we found that as children grow, they tend to spend more time eating and adopt increasingly regular eating schedules, a trend reflected in our findings. The need for nighttime feed declines for newborns as they age (69), and by 12 months of age, the need for nighttime feeding should no longer be necessary (39). However, our findings indicated that nearly half of children aged 12 to <36 months were still being offered post-midnight meals, which is most likely to be in the form of milk feeds. Research on meal timing in children is limited, but chrono nutrition, an emerging field of study within childhood nutrition, sheds light on key aspects of feeding behavior, including the timing of food intake (i.e., when meals are consumed) (21). There is increasing evidence that a later eating rhythm may contribute to a higher risk of overweight and obesity in children. Although few studies have examined this aspect of chrono nutrition, it has been suggested that late-evening eating may negatively impact cardiometabolic health, increasing the risk of weight gain and adverse lipid profiles later on in life (70).

The key strengths of our study include its large sample size of 1,307 caregivers, providing a robust representation of feeding practices within Singapore’s diverse population. Moreover, our study is unique in its comprehensive assessment of multiple dimensions of feeding, including variety, autonomy, mealtime setting, and timing. By analyzing these practices across different age groups, we have generated valuable insights into age-specific behaviors and developmental stages, thereby highlighting opportunities for targeted interventions to support healthy feeding practices.

Several limitations of our study should be acknowledged. First, the majority of caregivers surveyed were from the Chinese ethnic group with university-level education, which may introduce sampling bias and limit the generalizability of our findings to other ethnic and socioeconomic groups. Second, our study relied on retrospective self-reports from caregivers, making it susceptible to recall bias, especially when recalling specific foods and feeding practices over a typical week. Additionally, social desirability bias may have influenced caregivers to provide responses they deemed more socially acceptable; however, the anonymity of the survey helps mitigate this potential bias. Lastly, while our survey addressed established guidelines and key feeding domains, it did not capture certain extrinsic factors, such as caregiver stress, cultural beliefs, and family dynamics, which could also influence feeding practices. Furthermore, future studies should include ethnic minorities to ensure that the results are valid and applicable to a wider population, and longitudinal research will be essential to track feeding practices and eating behaviors over time.

## Conclusion

In conclusion, dietary variety was limited for some infants and toddlers, with the early introduction of processed foods and sugar-sweetened beverages being common. Feeding autonomy challenges, reduced caregiver responsiveness to hunger and satiety cues, and behaviors such as reliance on specific mealtime settings, screen use during meals, and continued post-midnight feedings in toddlers were also observed. These findings underscore the critical role of caregivers in shaping early childhood feeding practices across key domains. Our study reinforces the importance of the Singapore Guidelines for Eating and Feeding in Infants and Young Children (71) and highlights the need for culturally tailored interventions to enhance complementary feeding practices and expand caregiver knowledge across diverse, multi-ethnic populations. Future research should focus on developing and evaluating such interventions to improve feeding practices and nutritional outcomes.

## Data Availability

The raw data supporting the conclusions of this article will be made available by the authors upon reasonable request.

## Footnotes

^1^ https://form.gov.sg/
